# Multisession tDCS combined with intrastimulation training improves emotion recognition in adolescents with autism spectrum disorder

**DOI:** 10.1016/j.neurot.2024.e00460

**Published:** 2024-10-10

**Authors:** Karin Prillinger, Gabriel Amador de Lara, Manfred Klöbl, Rupert Lanzenberger, Paul L. Plener, Luise Poustka, Lilian Konicar, Stefan T. Radev

**Affiliations:** a Department of Child and Adolescent Psychiatry, Medical University of Vienna, 1090 Vienna, Austria; b Comprehensive Center for Pediatrics (CCP), Medical University of Vienna, 1090 Vienna, Austria; c Comprehensive Center for Clinical Neuroscience and Mental Health (C3NMH), Medical University of Vienna, 1090 Vienna, Austria; d Department of Psychiatry and Psychotherapy, Medical University of Vienna, 1090 Vienna, Austria; e Department of Child and Adolescent Psychiatry and Psychotherapy, University of Ulm, 89073 Ulm, Germany; f Department of Child and Adolescent Psychiatry, University Hospital Heidelberg, 69115 Heidelberg, Germany; g Cognitive Science Department, Rensselaer Polytechnic Institute, 12180 Troy, New York, USA; h Center for Modeling, Simulation and Imaging in Medicine (CEMSIM), Rensselaer Polytechnic Institute, 12180 Troy, New York, USA

**Keywords:** Autism spectrum disorder, Transcranial direct current stimulation, Social cognition, Intrastimulation training, Electric field simulation, Task activation patterns

## Abstract

Previous studies indicate that transcranial direct current stimulation (tDCS) is a promising emerging treatment option for autism spectrum disorder (ASD) and its efficacy could be augmented using concurrent training. However, no intrastimulation social cognition training for ASD has been developed so far. The objective of this two-armed, double-blind, randomized, sham-controlled clinical trial is to investigate the effects of tDCS combined with a newly developed intrastimulation social cognition training on adolescents with ASD. Twenty-two male adolescents with ASD were randomly assigned to receive 10 sessions of either anodal or sham tDCS at F3/right supraorbital region together with online intrastimulation training comprising basic and complex emotion recognition tasks. Using baseline magnetic resonance imaging data, individual electric field distributions were simulated, and brain activation patterns of the training tasks were analyzed. Additionally, questionnaires were administered at baseline and following the intervention. Compared to sham tDCS, anodal tDCS significantly improved dynamic emotion recognition over the course of the sessions. This task also showed the highest activations in face processing regions. Moreover, the improvement was associated with electric field density at the medial prefrontal cortex and social awareness in exploratory analyses. Both groups showed high tolerability and acceptability of tDCS, and significant improvement in overall ASD symptoms. Taken together, multisession tDCS improved dynamic emotion recognition in adolescents with ASD using a task that activates brain regions associated with the social brain network. The variability in the electric field might diminish tDCS effects and future studies should investigate individualized approaches.

## Graphical abstract

**Figure:**
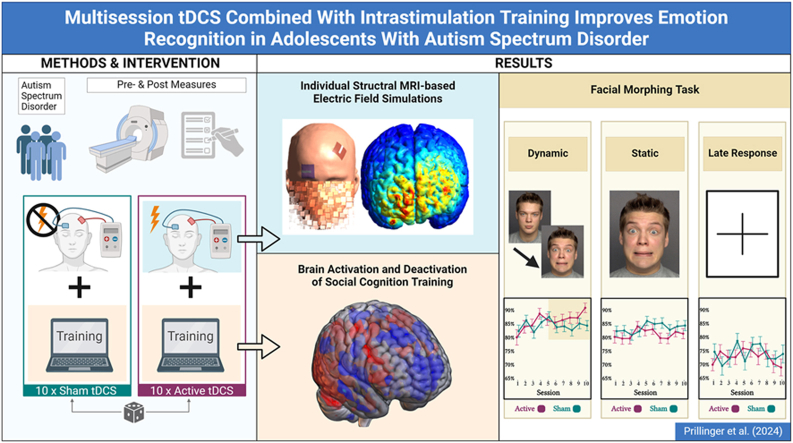

## Introduction

Transcranial direct current stimulation (tDCS) has been investigated as a tool for neuroenhancement in healthy individuals [1,2] and for therapy in patient populations [3]. Recently there have been encouraging findings regarding its effects on individuals with autism spectrum disorder (ASD) [4,5], a neurodevelopmental disorder with limited evidence-based treatment options [[6], [7], [8]]. A core feature of this heterogeneous disorder is impaired social functioning, which includes difficulties in decoding others’ emotional states from facial expressions [9,10]. Therapeutic tDCS for ASD typically comprises 1–2 ​mA for 20 ​min, with the anode placed over the left dorsolateral prefrontal cortex (dlPFC) being the recommended stimulation site [4,11]. This setup elicits peak current density at the medial prefrontal cortex (mPFC) with the common bipolar electrode montage [12,13]. The mPFC is suggested as an anodal stimulation target in ASD [5,14] as it has repeatedly been shown to be a key node for social cognition involved in cognitive and affective Theory of Mind and emotion recognition abilities [[15], [16], [17], [18], [19], [20], [21], [22]].

In individuals with ASD, atypical activity and functional connectivity within various brain regions and networks during social cognition tasks have been suggested as a strong neurobiological characteristic [[23], [24], [25], [26], [27]]. Specifically, the mPFC showed reduced activation during the performance of social cognition tasks in participants with ASD relative to typically developing controls [23,28,29]. Regarding functional connectivity, analyses revealed underconnectivity among several brain areas during social cognition tasks in participants with ASD. Especially, underconnectivity between frontal (such as mPFC) and posterior regions was reported [23,29]. In summary, these findings elucidate disturbances within the brain circuitry that underlies social cognition and provide a promising opportunity for intervention through neurophysiological techniques.

Anodal tDCS has the ability to increase cortical excitability and activity in the stimulated region as well as functional connectivity in the associated neural networks [30]. Thus, the application of anodal tDCS targeting the mPFC could be used to mitigate atypical brain activity and thereby enhance the participants’ social cognition abilities. Studies investigating tDCS targeting the mPFC reported its ability to influence neural activity and enhance emotion recognition abilities in healthy adults [31,32]. Furthermore, anodal tDCS over the mPFC enhanced social cognition skills [33] and increased mPFC activity [34]. Moreover, functional magnetic resonance imaging (fMRI) studies have shown that tDCS could modulate functional connectivity, such as between the mPFC and the default mode network (DMN) [35], as well as between the mPFC and anterior-insula [34].

MPFC activation could also be increased by trainings for facial emotion recognition and social cognition in children and adolescents with ASD [36,37]. As tDCS delivered to an “active” brain region as opposed to a “resting” one has repeatedly shown greater functional outcomes, combining tDCS and intrastimulation training has been increasingly investigated [[38], [39], [40], [41]]. Specifically, the effects of concurrent tDCS with physical (for a meta-analysis see Ref. [42]) and cognitive trainings have been extensively investigated and found to have synergistic effects. The predominant focus of these cognitive trainings involved different forms of working memory trainings, leading to enhancements in healthy adults [43], patients with cognitive impairment [44], adults with depression [41,45], and adolescents with Attention Deficit-/Hyperactivity Disorder [46]. Nevertheless, the majority of clinical trials investigating tDCS in ASD have administered the stimulation while patients were at rest (e.g., Refs. [47,48]) or did not report what participants were doing during the stimulation (e.g., Refs. [[49], [50], [51], [52]]). These previous findings suggest that intrastimulation training of social-emotional skills might enhance tDCS-induced improvements in individuals with ASD. However, no such computer-based training of social cognition in adolescents with ASD has been developed and evaluated so far [53].

Moreover, investigations seeking to understand which brain regions and networks are activated during intrastimulation training are lacking and could provide crucial insights into the neurophysiological mechanisms underlying tDCS-dependent behavioral changes. An additional factor influencing tDCS outcomes is the magnitude and distribution of the electric current reaching the targeted brain regions [54]. Individual anatomical factors, including skull thickness, brain morphology, and cerebrospinal fluid can alter the electric field (e-field) generated by tDCS, leading to variations in the intensity and focality of stimulation across individuals [55,56]. This individual e-field could play a critical role in determining the efficacy and variability of tDCS interventions [56,57], and recent studies have already reported an association between e-field strength and tDCS-induced changes in cognitive and behavioral outcomes [[58], [59], [60]]. However, no such studies exist in the context of tDCS interventions targeting ASD.

The current clinical trial aimed to examine the effects of tDCS, coupled with a self-developed intrastimulation social cognition training (previously described in Ref. [12]), on emotion recognition abilities in adolescents with ASD. To address this primary objective, we analyzed the differential effects of social cognition training during the stimulation sessions in both the active tDCS and sham tDCS groups. Additionally, to investigate potential stimulation factors associated with tDCS effects on social cognition, we performed individual simulations of the distribution of the e-field to quantify the e-field magnitude at our stimulation target (i.e., the mPFC). To assess task-specific engagement of brain regions, we investigated fMRI activation and deactivation patterns during the training tasks at baseline. Finally, to link behavioral outcomes with neurophysiological outcomes and ASD symptoms, we explored associations between changes in emotion recognition abilities with e-field magnitude and parent-reported changes in ASD symptoms.

## Material and methods

### Trial design

This randomized, double-blind, and sham-controlled clinical trial was conducted at the Department of Child and Adolescent Psychiatry at the Medical University of Vienna and in accordance with the declaration of Helsinki. The trial was registered in the German Registry of Clinical Trials (DRKS00017505) and approved by the Austrian Agency for Health and Food Safety and the ethics committee of the Medical University of Vienna (EK1624/2018). Written informed consent was obtained from all participants and caregivers involved in the study. This study comprised pre-MRI measures, pre- and post-measures of the caregiver-rated Social Responsiveness Scale (SRS) [61], as well as behavioral emotion recognition tasks (ERT) for the ten days of tDCS intervention and during the pre-MRI measures. Pre- and post-intervention measures were conducted within 7 days before and after the intervention, respectively. Regarding self-reported measures, participants answered the questionnaire of sensations related to transcranial electrical stimulation [62] directly after the last session to assess if they experienced any side effects and if they believed they received actual or sham stimulation.

### Participants: Eligibility, randomization, and blinding

Male adolescents between 12 and 18 years were eligible for study inclusion if they fulfilled the following criteria: IQ ​≥ ​70, right-handedness as assessed in the initial interview and with the Edinburgh Handedness Inventory [63], no prior experience with neurostimulation, no contraindication for tDCS or MRI, no concomitant psychopharmacological medication, no severe neurologic or psychiatric disorders or medical conditions (such as epilepsy or other seizure-related disorders, psychosis, skull defects, craniotomy, and the presence of medical devices such as cardiac pacemakers, defibrillators, cochlear implants, intracranial or cranial stimulators, or other metal implants in the head) and an ICD-10 diagnosis for ASD using Autism Diagnostic Observation Schedule (ADOS) [64] or Autism Diagnostic Interview (ADI-R) [65]. Eligible participants were assigned to either the experimental or the control group following a block randomization scheme. To guarantee a double-blind procedure, a code was used to operate the tDCS device, and a researcher not involved in any patient contact or device operation conducted group allocation and code preparation.

### Intervention: tDCS and intrastimulation social cognition training

The study involved ten sessions over two consecutive weeks. During each session, participants received either 20 ​min of anodal tDCS or sham stimulation. Whenever possible, all stimulation sessions were scheduled at the same time of day to minimize circadian rhythm effects [66]. Anodal tDCS was applied at 2 ​mA using an Eldith-DC Stimulator (NeuroConn GmbH, Germany) with 3.2 ​× ​3.2 ​cm rectangular rubber electrodes (NeuroConn GmbH, Germany) and Ten20 paste (Weaver and Company, USA) to hold the electrodes in place and prevent bridging. The anode was placed at F3 (following the 10–20 electroencephalography [EEG] system) using the Beam F3 method [67], and the cathode was positioned at the electrode position Fp2 over the right supraorbital region. Head circumference was measured, along with nasion-to-inion and tragus-to-tragus distances, to determine the Cz position as a reference point.

Stimulation began and ended with 30 ​s of fade-in/fade-out. For sham stimulation, 2 ​mA were applied for 40 ​s after the fade-in phase to replicate the initial skin sensation of active tDCS. During tDCS, all participants engaged in intrastimulation training. The training commenced immediately at stimulation onset and followed a standardized, computer-based procedure that ensured consistency across sessions while presenting different stimuli every session. For the first 5 ​min, participants watched scenes from the animated film “Inside Out” [68], which includes personifications of the five basic emotions. The movie was interrupted to ask participants questions targeting Theory of Mind and emotion recognition abilities as well as their own emotions watching the scenes. The movie segment was followed by the self-developed ERT [12,69], which is divided into three distinct explicit emotion recognition tasks: “*Face Emotion*”, “*Social Scenes*”, and “*Morphing*” (see Refs. [12,69] for a more detailed description and illustrations). While *Face Emotion* and *Social Scenes* contained longer videos of one or more actors expressing complex emotions, *Morphing* contained short videos of basic emotions and neutral expressions. All *Morphing* videos started with a neutral expression that morphed into a basic emotion within 1 ​s and remained static on the screen for another second before disappearing. Participants were instructed to interrupt the video as soon as they recognized the expressed emotion and select the correct emotion label from a list that appeared either 1) upon interruption or 2) automatically following the 2-s period. Dynamic *Morphing* is intended to capture more naturalistic emotion recognition, as facial emotions are often processed in fractions of a second [70]. The ERT utilized stimuli from validated databases representing individuals of different ages and genders [[71], [72], [73], [74]]. The stimulation ended with the next part of the question-interrupted movie. This self-developed procedure was intended to standardize the participants’ engagement and involve targeted brain networks during the stimulation administration. Additionally, it entailed an entertaining character for the adolescents.

### Task-induced activation and deactivation patterns

To assess which brain regions were involved in ERT execution at baseline, participants conducted the *Face Emotion* and the *Morphing* task within a 3T Siemens MAGNETOM Prisma MR Scanner (Siemens, Erlangen, Germany). Responses were collected using an MRI-compatible button box and inter-trial intervals of the ERT were MRI-adapted. Additionally, T1-weighted and T2-weighted MRI sequences were recorded. T1-weighted images were acquired using a turbo fast low angle shot (TFL) sequence with TE/TR/TI ​= ​2.29/2300/900 ​ms, FoV ​= ​165.44 ​× ​240 ​× ​240 ​mm, matrix size ​= ​256 ​× ​256, 176 slices, 0.94 ​× ​0.94 ​× ​0.94 ​mm voxel size (rounded), flip angle ​= ​8°, bandwidth ​= ​200 ​Hz/Px. T2-weighted images were acquired using a turbo spin echo (TSE) sequence with TE/TR ​= ​408/3,200 ​ms, FoV ​= ​172.8 ​× ​230 ​× ​230 ​mm, matrix size ​= ​256 ​× ​256, 192 slices, 0.9 ​× ​0.9 ​× ​0.9 ​mm voxel size (rounded), flip angle ​= ​120°, bandwidth ​= ​725 ​Hz/Px. During these structural scans, cartoons were shown to the participants. Sequence parameters and sample size calculations are in accordance with Prillinger et al., (2021) [12]. Preprocessing comprised physiological noise modeling and regression [75], slice-wise motion correction [76], slice-timing correction, realignment across measurements, normalization to a custom template [77] created using the CerebroMatic toolbox [78], wavelet despiking [79], and smoothing. For both tasks, the videos and answers were treated as separate conditions with one regressor containing all emotions. Nuisance regressors were modeled from white matter and cerebrospinal fluid using CompCor [80].

### Modeling of electric current flow

To assess the generated e-fields, SimNIBS 4.0 [81,82] was used. High-quality head models were generated for each participant in the active tDCS group using their T1-weighted and T2-weighted MRI from baseline measurements. The outlines of the reconstructed tissue compartments were visually inspected for accuracy. Afterward, the e-field was simulated using the above-described bilateral montage. Scripts were employed for a standardized simulation procedure, and after visual inspection, cathode placement was individually adapted if necessary. The input parameters encompassed electrode sizes (3.2 ​× ​3.2 ​cm square rubber electrodes with 1 ​mm thickness), Ten20 paste as conductor (2 ​mm gel thickness), and a rectangular connector positioned in relation to the electrode center.

### Outcome measures

In addition to structural and functional MRI, we collected behavioral, self-reported and caregiver-reported outcomes. For the behavioral outcomes, we analyzed the trial-by-trial recognition accuracy as the main outcome of the ERT. As *Morphing* is the only time-sensitive task, we also considered reaction time (RT) as a further factor.

To assess the effectiveness of participant blinding, participants answered a questionnaire about expected group assignments post-intervention, and two complementary blinding indices (BI) [83] were calculated. James' BI provides a summary measure covering both groups and regards “do not know” as the most important observation but does not give information about potentially different behaviors in the experimental and control groups. This is addressed by the more sensitive Bang's BI which puts more emphasis on the balance in proportions of correct vs. incorrect guesses and is interpreted as the percentage of unblinding beyond chance [83]. Additionally, participants were asked if they experienced any adverse effects during the stimulation. To assess blinding and side effects, an adapted German version of the questionnaire of sensations related to transcranial electrical stimulation was used at the last stimulation session [62].

The caregivers assessed changes in overall social functioning using the SRS [61]. The SRS measures the presence and severity of social impairment and consists of a total score and five subscales with higher scores indicating greater impairment. We analyzed the total score and the social awareness subscale, which comprises questions regarding the ability to recognize social cues [61].

### Statistical analysis

Statistical analyses were conducted using R, Version 4.1.3 [84], and the lme4 package [85]. Generalized linear mixed models (GLMMs) were formulated and fit to the trial-by-trial data of all three ERT, with binary Accuracy *(Correct vs. Incorrect)* as the outcome, Group *(Control vs. Experimental)*, Session [[1], [2], [3], [4], [5], [6], [7], [8], [9], [10]]*,* and their interaction as fixed effects, Occasion *(Offline vs. Online)* as a fixed effect with no interactions (i.e., a control variable), and participant identifier (ID) as a random effect. Since the outcome variable is binary, a Bernoulli distribution with a logit link function was used. The trial-by-trial analysis was conducted to avoid loss of information through data aggregation. For the analysis of the *Morphing* task, RTs were grouped into three conditions (dynamic, static, late) based on the stimuli phase in which the participants responded. The dynamic and static conditions include all responses provided in the dynamic (morphing) and static phases, respectively. The late condition includes all answers recorded after the 2-s time window when the stimulus had already disappeared. Importantly, this response condition was added as a *moderator* in the *Morphing* analysis to account for different group-session interactions based on the timing of responses. This coarse discretization of RTs was performed to include all individual trial outcomes in the analysis, irrespective of whether a participant was able to interrupt the video within the 2-s on-screen period or not.

To determine the effects of the intervention, we report likelihood ratio tests between each full model (i.e., containing all main effects and interactions) and a corresponding reduced model featuring no *Group* factor as a predictor. We also consider Bonferroni-corrected *p*-values of main effects and interactions statistically significant at the conventional confidence level of 0.05. The correction was performed to account for multiple testing resulting from having one GLMM per task, resulting in a corrected confidence level of 0.0167.

Preprocessing, modeling, and testing of MRI data were mainly conducted using Statistical Parametric Mapping, version 12 [86]. Baseline activation of the ERTs was estimated via one-sample t-tests in SPM12. Additionally, a comparison of the whole-brain baseline activation of both tasks was performed using the pre-treatment fMRI scans of all participants via a paired *t*-test.

The blinding indices were analyzed according to Bang et al. (2010) [83] and side events with the Likelihood Ratio Test. To examine the effects of tDCS on social functioning, we analyzed the SRS total and subscale scores using Group (*Control vs. Experimental*) x Time (*Pre vs. Post*) repeated-measures analysis of variance. Exploratory analyses aiming to elucidate potential relationships between behavioral improvements and both subjective social awareness and the intensity of cortical stimulation were conducted. Accordingly, Spearman's rank order and Pearson correlation coefficients were computed to examine the associations between improvements in the dynamic Morphing segment and changes in the social awareness subscale, as well as mean e-field at the mPFC.

## Results

In total, 23 participants were included in the trial, with one participant having to be excluded after the fourth session due to COVID-19 restrictions. The 22 participants (mean age 14.1 ​± ​1.9 years) receiving all ten tDCS sessions and conducting all baseline- and post-measurements were included in the analyses (for Consort Flow Diagram see Prillinger et al., (2023) [69]). The performance scores of the ERT, SRS scores, and age of both groups are presented in (Table 1).

**Table 1:** Performance scores.

	Active				Sham			
	Pre		Post		Pre		Post	
	Mean	SD	Mean	SD	Mean	SD	Mean	SD
Age	14.00	1.90	–	–	14.27	1.90	–	–
SRS total score	96.82	28.80	78.55	23.60	97	34.89	77.56	28.02
SRS social awareness	12.82	4.94	10.46	4.44	10.91	4.51	9.91	4.16
	Session 1		Session 10		Session 1		Session 10	
Morphing-dynamic	0.80		0.91		0.82		0.84	
Morphing-static	0.80		0.81		0.82		0.84	
Morphing-late response	0.70		0.69		0.75		0.74	
Social scenes	0.68		0.83		0.81		0.83	
Face emotion	0.64		0.64		0.69		0.76	

Note: Higher scores in the SRS indicate greater impairment, whereas higher values in *Morphing*, *Social Scenes*, and *Face Emotion* indicate higher accuracy.

### Intrastimulation social cognition training

The Occasion (Online vs. Offline) was not a significant predictor of accuracy in any of the three tasks (*Morphing*: OR ​= ​0.93, 95%-CI [0.86–1.00], *p* ​= ​.067*; Social Scenes*: OR ​= ​1.08, 95%-CI [0.92–1.28] *p* ​= ​.358; *Face Emotion*: OR ​= ​0.94, 95%-CI [0.85–1.04], *p* ​= ​.226). Regardless of significance, we kept Occasion as control variable in all models.

#### Morphing

**Fig. 1:**
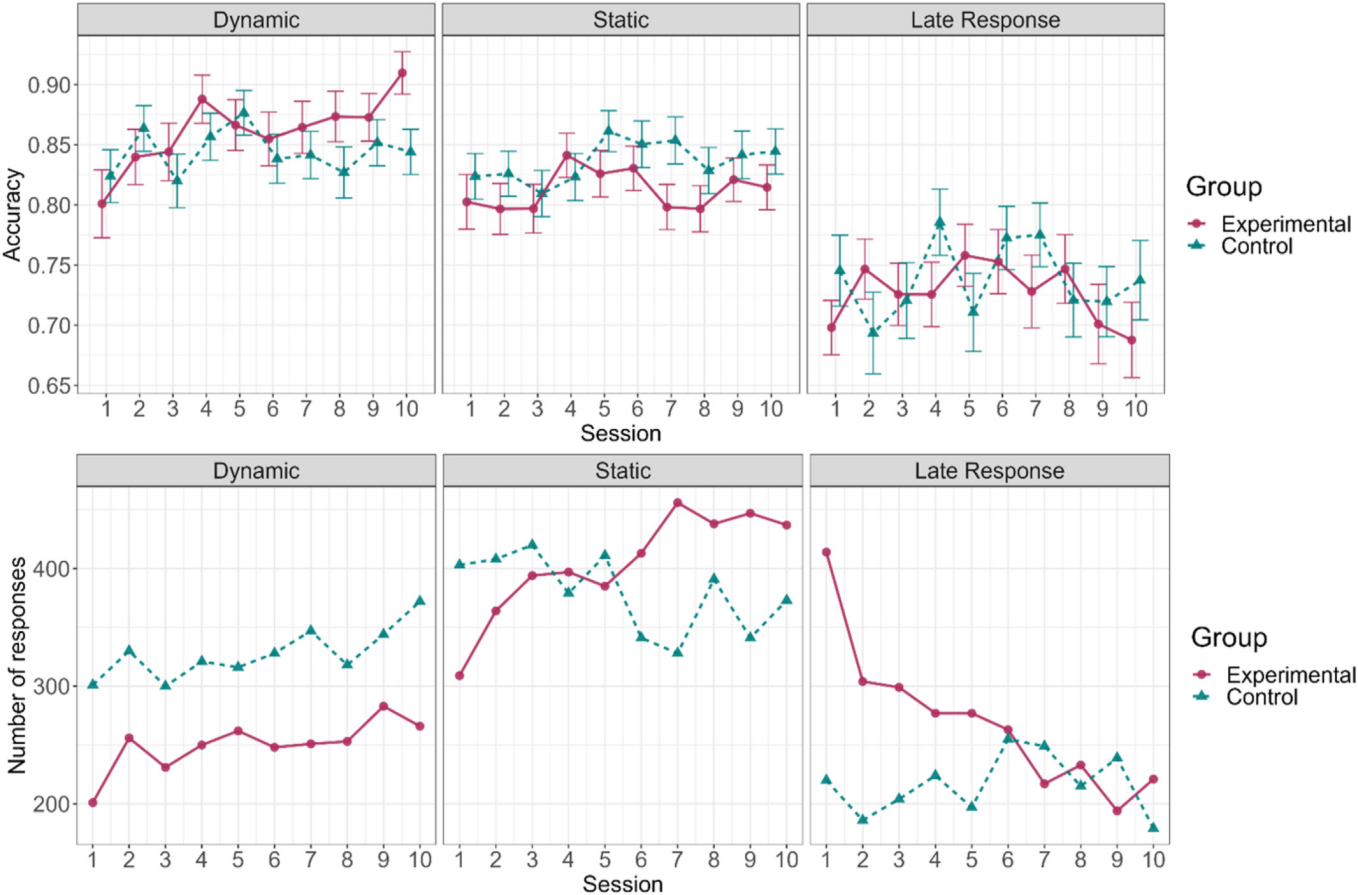
*Morphing* Task. *Top row*: Average accuracy across all training sessions for both groups and all three task segments. The most notable improvement in accuracy occurs for responses in the dynamic time window (first column). Error bars depict standard error of the mean (SEM) to highlight the estimation uncertainty due to different numbers of responses in each task segment. *Bottom row:* Total number of responses in each task segment across all training sessions. The number of late responses in the experimental group drastically decreases, leading to more responses in the dynamic and static segments.

The likelihood ratio test between the full model and the reduced model resulted in a significant preference for the full model, χ2 [6] ​= ​17.48, *p* ​= ​.008, warranting the inclusion of the Group factor. The full model estimated significant main effects of Condition, OR ​= ​0.54, 95%-CI [0.42–0.70], *p* ​< ​.001 and OR ​= ​0.44, 95%-CI [0.31–0.63], *p* ​< ​.001 for the Static and Late conditions (relative to the Dynamic condition), respectively. These were accompanied by a significant two-way interaction between Session and Group, OR ​= ​1.07, 95%-CI [1.02–1.13], *p* ​= ​.011, implying that the experimental group showed a larger improvement in recognizing the stimuli during the dynamic phase compared to the control group. Such differential improvements were not present in the other conditions. The aggregated data underlying the above hierarchical model are presented in Fig. 1, top row. The auxiliary bottom row of the figure also displays that the participants in the experimental group markedly increased the number of their responses in the Dynamic and Static conditions relative to the Late condition. This pattern underscores that the improved dynamic recognition performance of participants in the experimental group was not due to a drop in their response rate to dynamic stimuli. Note, that our trial-by-trial analysis does not aggregate the data, so differences in trial numbers across different factors are automatically encoded in the estimates.

#### Social Scenes

**Fig. 2:**
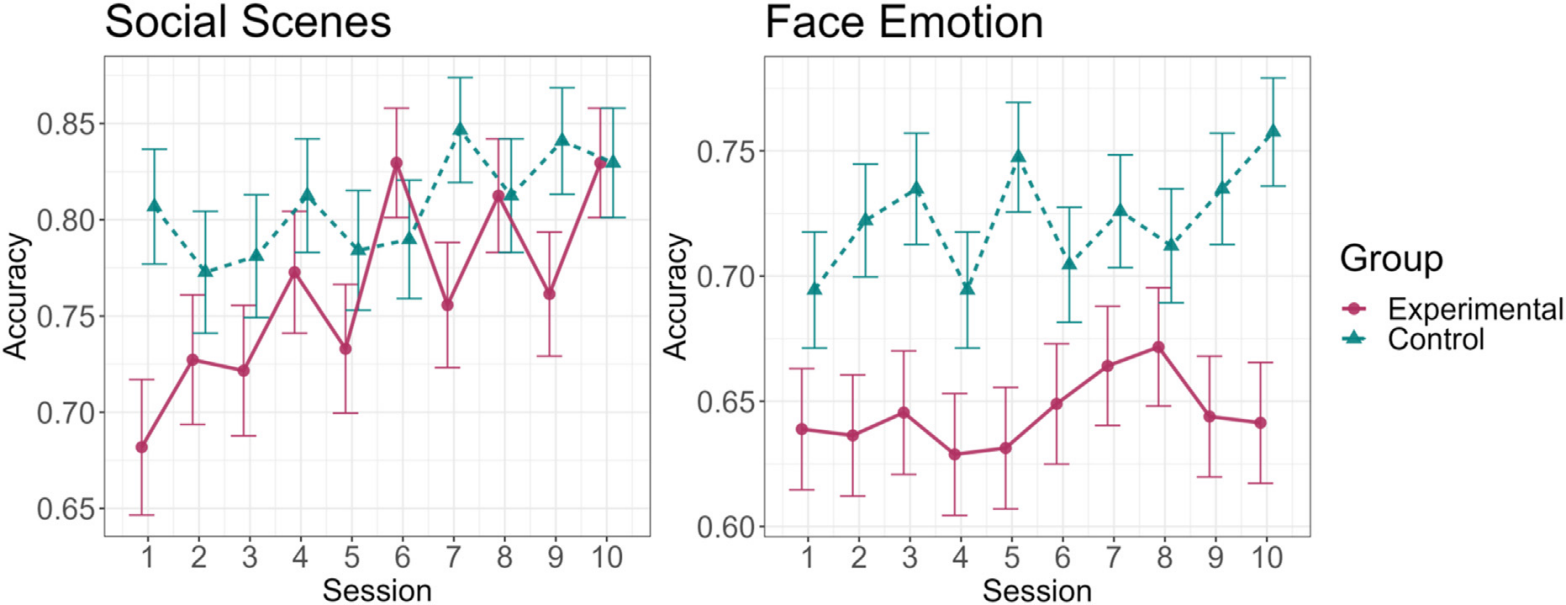
*Left graphic*: An increase in accuracy over the course of the training in the *Social Scenes* task in both groups. Still, the experimental group exhibits a visibly steeper improvement given notable baseline differences. *Right graphic:* No significant changes in task performance in the *Face Emotion* task across the training in both groups.

The likelihood ratio test between the full model and the reduced model did not yield a significant result, χ2 [2] ​= ​2.33, *p* ​= ​.31, leading to the selection of the reduced model without a Group factor. This model estimated a significant main effect of Session, OR ​= ​1.06, 95%-CI [1.03–1.09], *p* ​< ​.001, suggesting an overall increase in accuracy over the course of the training (see Fig. 2).

#### Face Emotion

The likelihood ratio test between the full model and the reduced model did not yield a significant result, χ2 [2] ​= ​2.26, *p* ​= ​.32, and there were no significant effects in either of the models. A likelihood ratio test between the reduced model and an intercept-only model (i.e., having no fixed effects) yielded a non-significant effect, χ2 [2] ​= ​3.90, *p* ​= ​.14, suggesting no systematic changes in task performance across the training (see Fig. 2).

### Simulation of electric fields and ROI analysis of mPFC

**Fig. 3:**
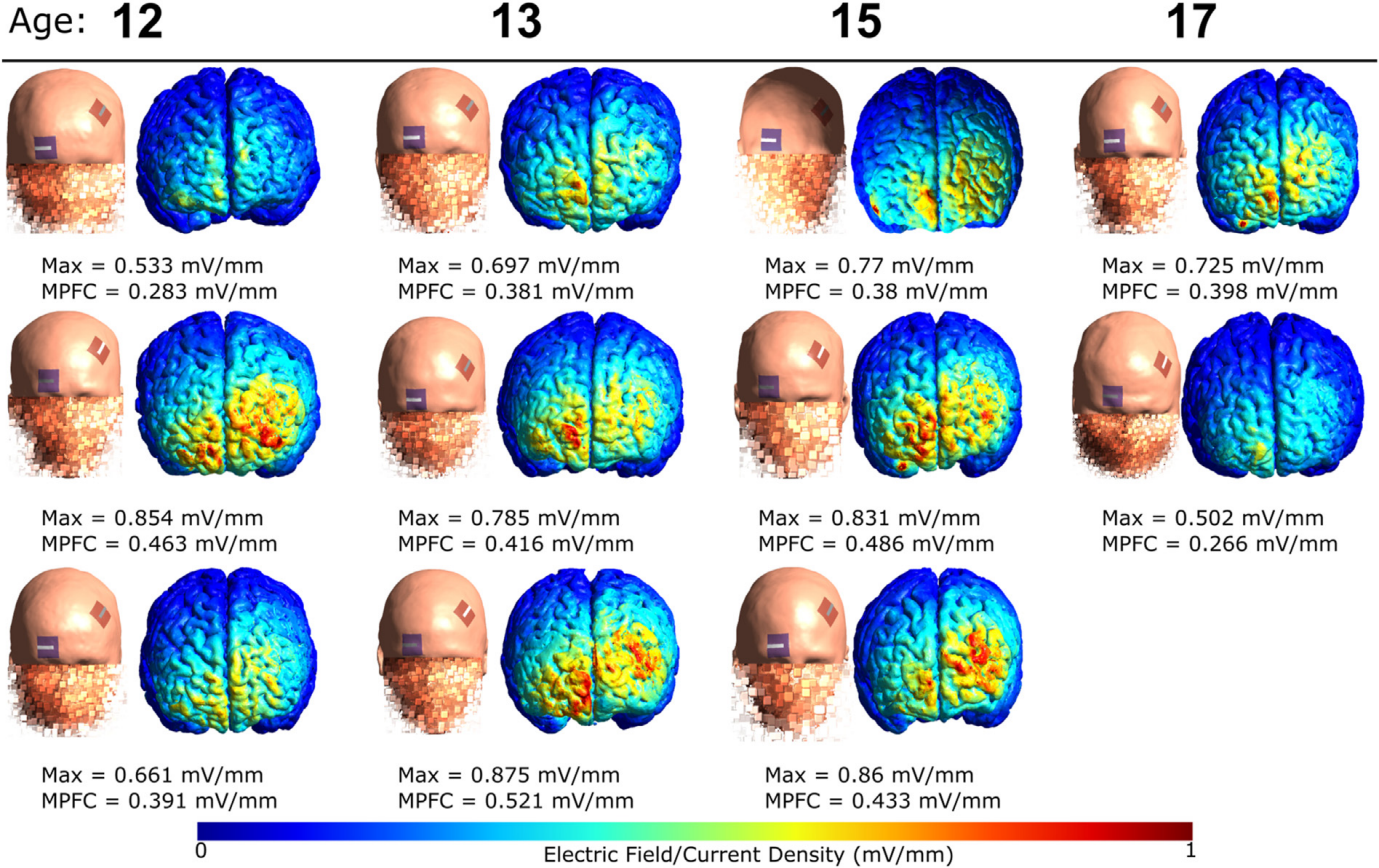
Peak magnitudes of the intracranially generated e-fields and mean e-fields of the mPFC are listed below each simulated configuration for the eleven participants of the active tDCS group. Note: Max ​= ​overall peak magnitude of e-field, mPFC ​= ​mean e-field at medial prefrontal cortex.

The gray matter surface with the magnitude of the e-fields and the individual peak values are illustrated in Fig. 3. In addition, based on the e-field simulations, ROI means of the e-fields of our stimulation target, the mPFC, were calculated for every participant in the active tDCS group using the MNI coordinates [−2, 58, 20] and 6-mm spherical radius [14] (see Fig. 3).

### Activation and deactivation during task conduction

**Fig. 4:**
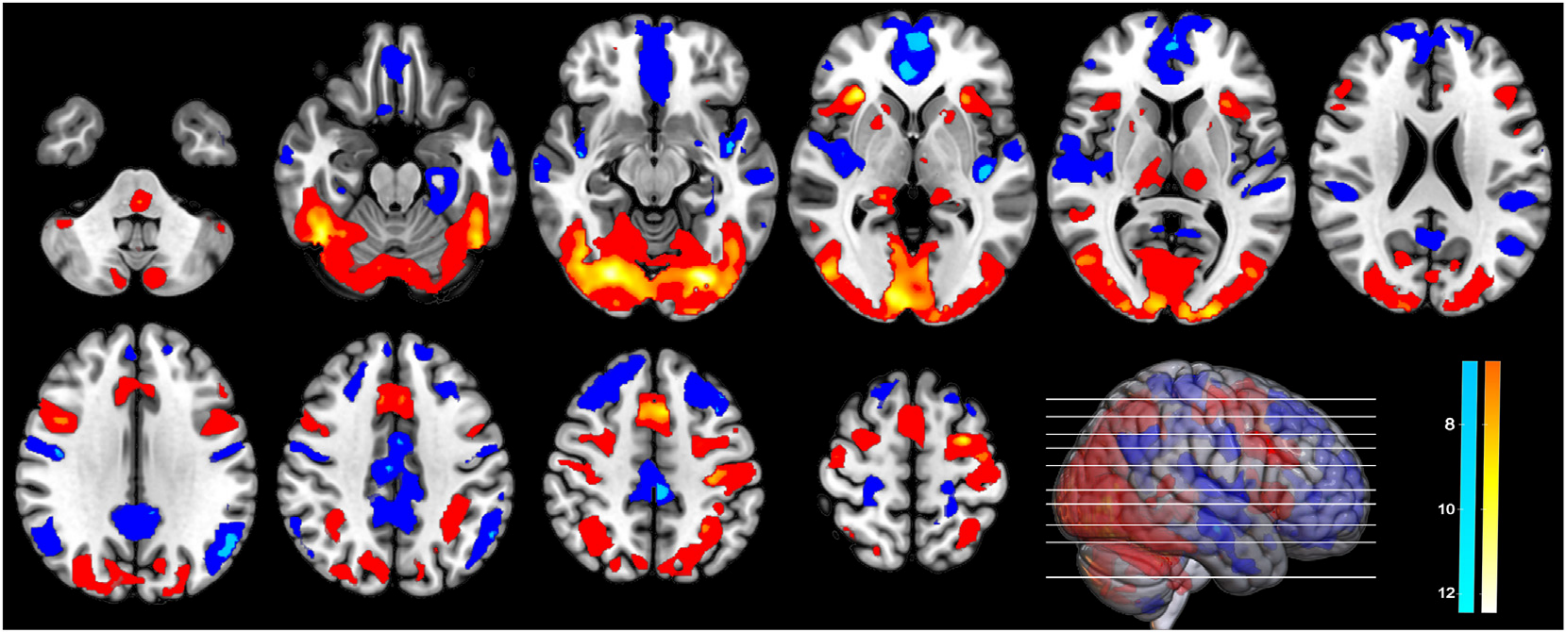
Activation (red-yellow) and deactivation (blue-cyan) pattern in the *Morphing* task at baseline; slices displayed: z ​= ​−42, −22, −12, 0, 8, 22, 32, 40, 50, 60 ​mm in the coordinate system defined by the Montreal Neurological Institute. Uniform colors represent significant clusters and color gradients peak-level effects.

All significant activation and deactivation peak- and cluster-level effects of the *Face Emotion* and *Morphing* task conducted during fMRI acquisition were visually inspected. Using the functional network parcellation defined by Yeo et al. (2011) [87], we found the following activation and deactivation patterns during the *Morphing* task (see Fig. 4). There was a general deactivation across the components of the DMN [88]. Within the dorsal attention network, there was activation in the intraparietal sulcus, which is also part of the social brain network [89]. Within the frontoparietal network, there was activation around another part of the social brain network [90] the inferior frontal sulcus, which is situated between the dlPFC and ventrolateral PFC and thereby affected by the tDCS-induced e-field. Within the ventral attention network, activation in the cingulate sulcus extending to the supplementary motor area as well as activation in the anterior insula, which is part of the social brain network, was found [90]. Regarding the basal ganglia, there was activation in the posterior part of the hippocampus which is involved in emotional processing [[90], [91], [92]]. The visual network was largely activated, including the fusiform face area which is implicated in facial processing [93,94]. Tables with significant peak coordinates and a summary illustration for the *Face Emotion* task are provided in the supplement (Fig. S1, Tables S1 and S2).

**Fig. 5:**
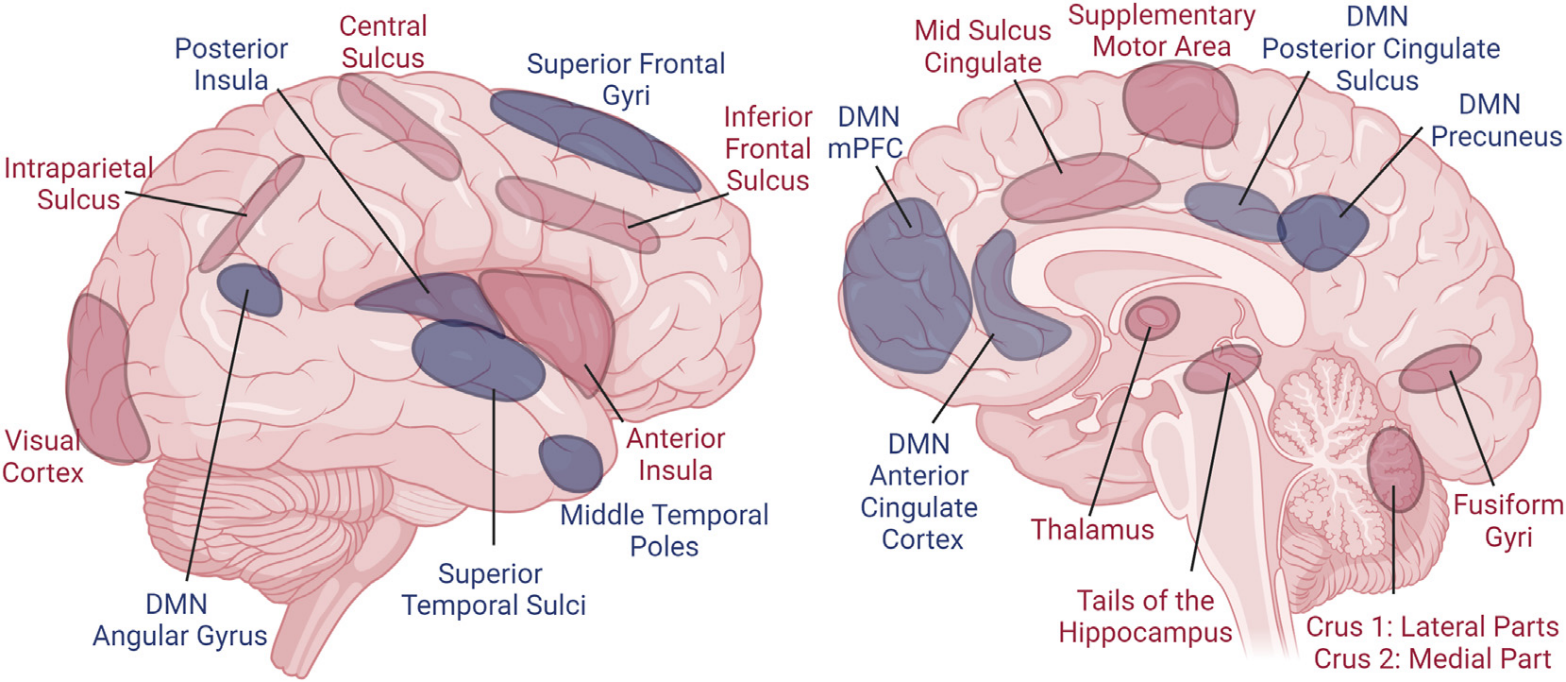
Regions with more activation (in red) and deactivation (in blue) in the *Morphing* compared to the *Face Emotion* task. DMN ​= ​Default Mode Network.

Comparing whole-brain activation in the *Morphing* and *Face Emotion* tasks, we found that the *Morphing* task showed a significantly stronger activation throughout the visual cortex, the fusiform gyri, the intraparietal sulcus, the supplementary motor area, along the inferior frontal and central sulci, the mid cingulate sulcus, the anterior insula, the thalamus, the tails of the hippocampus, and parts of the cerebellum (lateral parts of crus 1 and medial part of crus 2). Furthermore, *Morphing* showed significantly stronger deactivation in the superior frontal gyri, the posterior insula, the superior temporal sulci, and middle temporal poles as well as throughout the DMN including the anterior and posterior cingulate sulci, angular gyrus, the mPFC, and the precuneus (see Fig. 5).

### Clinical measures & exploratory analyses

There was a significant main effect of time for the SRS total score, *F*(*2.12, 42.44*) ​= ​*32.69*, *p* ​< ​*.001,* and social awareness subscale, *F*(*2.34, 46.84*) ​= ​*15.53*, *p* ​< ​*.001.* As further exploratory analyses, Spearman's rank order and Pearson correlations indicated a moderate to high relationship between the improvement in the dynamic *Morphing* segment and the change in the social awareness subscale of the SRS, *ρ* ​= ​.41, *r* ​= ​0.67. Similarly, a moderate relationship between the improvement in the dynamic *Morphing* segment and the mean e-field at the mPFC in the active tDCS group was observed, *ρ* ​= ​.38, *r* ​= ​0.47.

### Blinding, tolerability, acceptability

Based on James' BI, blinding was successful in all cases. Bang's BI, however, showed that 36 % of participants could be unblinded in the sham tDCS group and blinding was only successful in the active tDCS group (see Table S3 in the supplement).

Acceptability of the tDCS intervention was very good, as the only dropout was due to Covid-19 restrictions, all other participants completed all sessions and measurements. In terms of tolerability, none of our participants experienced a severe side effect. However, several participants reported mild and transient side effects, which were more common at the beginning of the stimulation and in general more in the active than in the sham condition (see Table S4 in the supplement).

## Discussion

This clinical trial is the first investigation of multisession tDCS augmented with social cognition intrastimulation training in adolescents with ASD. Specifically, we evaluated session-by-session changes of emotion recognition abilities and controlled for online (intrastimulation) and offline (pre-stimulation) effects. Moreover, we investigated potential neurophysiological factors underlying the effects by 1) assessing and comparing brain activation and deactivation patterns involved in the ERT and 2) modeling individual e-field densities. The goal of this intervention tailored to the social cognition construct was to directly impact impaired functions that hinder patients with ASD in daily life. Further, it aimed to explore therapeutic means to improve ASD by modulating the excitability of the targeted dysfunctional neural networks.

We found that tDCS targeting the mPFC and our social cognition training, but not sham tDCS with the training, significantly improved naturalistic emotion recognition abilities in individuals with ASD. Changes in emotion recognition were associated with peak e-fields at the mPFC and improved social awareness in exploratory analyses.

However, the improvement in the social cognition training was not uniform across all tasks. While in the *Morphing* task, accuracy increased for the active tDCS group during the time-sensitive dynamic phase, both groups improved in the *Social Scenes* task, but no changes were seen in the *Face Emotion* task. The latter two differed from the *Morphing* task, as the emotions were expressed continuously for at least nine and five ​s, respectively, without a morphing process or a time-sensitive component (i.e., participants had to interrupt the stimuli as soon as they recognized the emotion). In addition, these two tasks entailed the recognition of complex emotions, which are more influenced by context and culture, and are typically more challenging for individuals with ASD to recognize than basic emotions [95,96].

Taken together, brief image presentation and restricted response time, which increase task difficulty [97], in conjunction with relatively less challenging stimuli representing basic emotions, differentiated the *Morphing* task from the other ERT tasks. Thus, differences in cognitive demand due to the specific task characteristics could be responsible for the performance improvement of the experimental group only in the dynamic aspect of the *Morphing* task. Interestingly another study investigating children with ASD, also reported that improvements in social cognition tasks depended on the difficulty level of the task [98]. This study examined anodal tDCS targeting the ventromedial PFC, combined with a Theory of Mind test, yielding significant improvements only in precursor abilities and elementary, but not advanced, Theory of Mind [98]. Moreover, a study investigating anodal tDCS of the left dlPFC, combined with working memory paradigms with different task difficulties, reported that post-intervention improvement depended on the cognitive demands of the intrastimulation task [99].

It has been postulated that combining tDCS with cognitive tasks could lead to targeted changes in brain activity, contingent on how effectively the intrastimulation tasks engage relevant cognitive domains and brain networks [99]. While for other tasks, such as working memory n-back tasks, robust network engagement and cognitive load-dependency have been demonstrated [100,101], similar research on social cognition tasks in adolescents with ASD is notably absent. To address this, we implemented a two-stage process. First, to test if our tasks elicited the intended neural engagement, we investigated which networks and regions were active during the *Morphing* and *Face Emotion* task. Our intrastimulation *Morphing* task activated several regions involved in the social brain network such as intraparietal sulcus, inferior frontal sulcus, cingulate sulcus, anterior insula, hippocampus, and fusiform face area. As expected, our task deactivated all components of the DMN, including the mPFC, which is a core region of the DMN [102] and our stimulation target. The defining characteristic of the DMN is its deactivation during engaging activities. This deactivation is functionally important for goal-directed, externally-oriented cognition and could improve subsequent retrieval of learned information [103]. The activation of the mPFC due to social cues might thus be covered by the DMN-related deactivation of the region, resulting in a net deactivation of the mPFC during the intrastimulation task.

Second, to understand how the cognitive demands of different versions of social cognition tasks are reflected in the brain, we compared the activation patterns of the *Morphing* and *Face Emotion* tasks. Overall, the *Morphing* task showed significantly stronger activation and deactivation. In particular, significantly stronger activations in almost all regions of the face processing network [104] and several regions of the social brain network [90] suggest more engagement of these regions leading to specific training effects. The comparison of the *Morphing* and *Face Emotion* tasks also revealed increased activation in the central sulcus, crucial for integrating sensory feedback and motor commands [105,106] as well as the thalamus, the hippocampus, and the cerebellar crus I/II, all implied in emotion processing [[90], [91], [92],^107^,^108^]. Additionally, the *Morphing* task showed significantly stronger deactivation throughout the DMN. These findings indicate that the *Morphing* task demanded more cognitive engagement and attention because higher cognitive demand often leads to reduced activity in the DMN as the brain shifts resources to specific task-related networks [109,110].

Overall, our preliminary results align with the activity-selectivity hypothesis, which assumes that the sub-threshold shifts in the probability of neuronal firing induced by tDCS may preferentially affect active networks of neurons compared to networks that are less active or at rest [99,111]. We posit that during the dynamic phase of the *Morphing* task, the relevant brain regions were sufficiently engaged, reaching the necessary cognitive demand for effective tDCS modulation. However, for the other tasks, this level of functional targeting was not achieved, possibly due to reliance on different cognitive processes and engagement of broader networks for the processing of complex emotions [112,113]. These networks are more spatially distributed and exhibit lower ‘goodness of fit’ for our tDCS set-up, potentially resulting in insufficient neuronal activity levels for tDCS to be effective [111,114].

A further factor influencing tDCS outcomes is the induced cortical e-field [115]. Given the relatively low focality of the induced e-field, it is possible that regions associated with working memory, such as the left DLPFC, were indirectly affected by the stimulation [1,116,117]. However, as our intrastimulation training was not designed to engage or measure working memory, it is unlikely that working memory alone accounts for the observed effects on social cognition. Future studies employing this electrode montage should consider incorporating specific assessments of working memory to further explore its potential role in these effects. To our knowledge, this is the first trial providing individual simulations of all participants with ASD receiving active tDCS. Within our sample, we found a high inter-subject variability regarding the e-field distribution, with a mean e-field at the mPFC ranging from 0.266 to 0.521 ​mV/mm, which might be associated with improvements in emotion recognition. While this result needs to be further investigated in larger trials, it is in line with studies suggesting a positive relationship between the magnitude of the simulated current density and tDCS-induced outcomes such as neurophysiological effects [54], functional connectivity 58 and behavioral changes [115,118]. While the high inter-subject variability can be partly explained by anatomical differences, which are even more pronounced in developing brains [56,57], the ideal cortical e-field for a neurophysiological or behavioral change remains unclear [119]. Individual dosing approaches using head-circumference [120] have been investigated and could provide a less time-consuming and cost-intensive way to implement individual dosing in clinical trials and thus improve the therapeutic potential.

Compared to a previous study [121], our trial did not find tDCS-specific improvements in the parent-reported SRS, as both groups significantly improved after the intervention. Notably, the prior study did not find tDCS-induced improvements in their intra-stimulation cognitive training [122]. Parameters differed regarding applied current and electrode size. Contrary to other trials, we used smaller electrodes and conductive paste instead of saline, which can lead to a more focal e-field magnitude and induce less diffuse e-fields [12]. Thus, less cortical, and subcortical areas might have been affected than in previous studies. In general, widely used and established outcome measures assessing the effectiveness of interventions for individuals with ASD are lacking [123], which further complicates the comparability with other tDCS in ASD studies using different outcome measures for ASD symptoms (e.g., Refs. [47,49,50,52,124]).

Finally, we presented two complementary blinding measures, of which Bang's BI suggested unblinding in the control group [83]. However, as we did not have a subjective participant-based outcome measure, possible unblinding in the control group should not be very influential regarding the results.

The preliminary findings of this clinical trial should be interpreted considering some limitations and suggestions for future research. As the sample size was relatively small and included only males, studies with larger and more diverse samples are required. Future studies should also administer a questionnaire to investigate the effectiveness of blinding the caregivers and the investigators involved in the trial. In addition, long-term clinical and behavioral measures should be included for a more comprehensive evaluation of tDCS augmented with cognitive-emotional trainings as a clinical treatment. Since we found improvements in social functioning in both groups, future studies could additionally examine a tDCS-only and a social cognition training-only group to better assess the effects of each intervention.

In conclusion, this double-blind, randomized controlled trial was conducted to investigate the effects and underlying neurophysiological mechanisms of multisession 2 ​mA tDCS with left dlPFC anode placement and right supraorbital region cathode placement delivered with concurrent computerized social cognition training in 12–18-year-old individuals with ASD. The results indicate that our protocol was a safe, feasible, and well-tolerated treatment for male adolescents with ASD and that it could promote their emotion recognition ability depending on task characteristics. We propose that in our study endogenous (social cognition training) and exogenous (anodal tDCS) modulation of the mPFC activity and associated networks interacted synergistically to elicit behavioral improvement. Future trials investigating the implications and parameters of intrastimulation trainings and applying individualized stimulation dosage would be beneficial to expand our understanding of the neurophysiological mechanisms of tDCS-induced changes in ASD.

## Trial registration

The trial was registered in the German Registry of Clinical Trials (DRKS00017505) on 02/07/2019.

## Funding

K.P., G.A., and S.T.R. were partly funded by the AUSTRIAN SCIENCE FUND (FWF), grant number KLI600B27. Open access funding was provided by Medical University of Vienna.

## Author Contributions

Conceptualization: K.P., G.A., M.K., L.P., L.K., and S.T.R.; Methodology: K.P., G.A., and S.T.R.; Software: K.P. and S.T.R.; Validation: G.A., M.K., R.L., P.P., L.P., L.K., and S.T.R.; Formal analysis: K.P., M.K., and S.T.R.; Investigation: K.P., G.A., and M.K.; Resources: R.L., P.P., L.P., L.K., and S.T.R.; Data Curation: K.P. and S.T.R.; Writing—Original Draft: K.P.; Writing—Review & Editing: S.T.R., G.A., M.K., R.L., P.P., L.P., L.K., and K.P.; Visualization: K.P.; Supervision: R.L, P.P., and L.P.; Project administration: K.P.; Funding acquisition: L.K. All authors read and agreed to the final version of the manuscript.

## Declaration of competing interest

The authors declare the following financial interests/personal relationships which may be considered as potential competing interests: Karin Prillinger reports financial support was provided by Austrian Science Fund. Gabriel Amador de Lara reports financial support was provided by Austrian Science Fund. Stefan T. Radev reports financial support was provided by Austrian Science Fund. If there are other authors, they declare that they have no known competing financial interests or personal relationships that could have appeared to influence the work reported in this paper.
