# Supplementary material for: Multisession tDCS combined with intrastimulation training improves emotion recognition in adolescents with autism spectrum disorder
Source: Neurotherapeutics. 2024 Oct 10;21(6):e00460. doi: 10.1016/j.neurot.2024.e00460 (PMC11585900; doi:10.1016/j.neurot.2024.e00460)
Supplement: Multimedia component 1 [file mmc1.docx]

**Multisession Transcranial Direct Current Stimulation Combined With Intrastimulation Training Improves Naturalistic Emotion Recognition in Adolescents With Autism Spectrum Disorder**

Supplement

Activation and Deactivation during task conduction

**Table S1**

Coordinates for Morphing task.

| Morphing |  | MNI coordinates | | | Anatomical brain region (MRIcroGL REF) AICHA |
| --- | --- | --- | --- | --- | --- |
| Activation | Peak |  |  |  |  |
|  |  | -46 | -78 | 12 | G_Occipital_Mid |
|  |  | -46 | -86 | 4 | G_Occipital_Lat-565 |
|  |  | -38 | -14 | 60 | S_Rolando-335 |
|  |  | -32 | -24 | 50 | S_Rolando-335 |
|  |  | -30 | 22 | 0 | G_Insula-anterior-376 |
|  |  | -28 | -2 | 60 | S_Sup_Frontal-610 |
|  |  | -28 | -52 | 48 | S_Intraparietal-358 |
|  |  | -20 | -30 | -2 | G_Hippocampus-2161 |
|  |  | -8 | -74 | -22 | Cerebellum |
|  |  | -6 | 14 | 48 | S_Cingulate-2116 |
|  |  | -2 | -36 | -42 | Medulla |
|  |  | 20 | -30 | -6 | G_ParaHippocampal-4165 |
|  |  | 28 | 0 | 52 | S_Sup_Frontal-610 |
|  |  | 32 | 26 | 0 | G_Insula_anterior-376 |
|  |  | 36 | -82 | 8 | G_Occipital_Lat |
|  |  | 36 | -54 | 52 | S_Intraparietal-257 |
|  |  | 42 | -74 | -16 | G_Occipital_Lat-363 |
|  |  | 42 | -12 | 58 | S_Rolando-335 |
|  |  | 44 | 8 | 32 | S_Inf_Frontal-217 |
|  |  | 52 | 14 | 38 | S_Inf_Frontal-217 |
| Activation | Cluster |  |  |  |  |
|  |  | -30 | 22 | 0 | G_Insula_anterior-376 |
|  |  | -6 | 14 | 48 | S_Cingulate-2116 |
|  |  | -2 | -36 | -42 | Medulla |
|  |  | 32 | 26 | 0 | G_Insula_anterior-376 |
|  |  | 36 | -54 | 52 | S_Intraparietal-257 |
|  |  | 42 | -74 | -16 | G_Occipital_Lat-363 |
|  |  | 44 | 8 | 32 | S_Inf_Frontal-217 |
| Deactivation | Peak |  |  |  |  |
|  |  | -52 | -64 | 30 | S_Sup_Temporal-590 |
|  |  | -44 | -20 | -2 | G_Temporal_Sup-283 |
|  |  | -44 | -6 | -14 | G_Insula-anterior-578 |
|  |  | -38 | 24 | 50 | G_Frontal_Mid-414 |
|  |  | -36 | 14 | 50 | G_Frontal_Mid-414 |
|  |  | -34 | 20 | 58 | S_Sup_Frontal-48 |
|  |  | -8 | -44 | 30 | G_Precuneus-3136 |
|  |  | -6 | -34 | 46 | S_Cingulate-6120 |
|  |  | -6 | 56 | 0 | S_Anterior_Rostral-1108 |
|  |  | -4 | -52 | 36 | G_Precuneus-3136 |
|  |  | -2 | -4 | 40 | S_Cingulate-5119 |
|  |  | 2 | -18 | 42 | G_Cingulum_Mid-3126 |
|  |  | 4 | 40 | 0 | G_Cingulum_Ant-1122 |
|  |  | 6 | -56 | 20 | G_Precuneus-2135 |
|  |  | 10 | -48 | 38 | G_Cingulum_Post-2128 |
|  |  | 12 | 52 | 2 | S_Anterior Rostral-1108 |
|  |  | 20 | 64 | 20 | G_Frontal_Sup-23 |
|  |  | 24 | -28 | 64 | S_Rolando-436 |
|  |  | 40 | -10 | 2 | G_Temporal_Sup-283 |
|  |  | 42 | -10 | -12 | G_Insula_anterior-578 |
|  |  | 42 | -10 | 36 | S_Rolando-133 |
|  |  | 50 | -32 | 22 | G_Supramarginal-145 |
|  |  | 54 | -60 | 26 | S_Sup_Temporal-590 |
|  |  | 54 | -20 | 6 | G_Temporal_Sup-384 |
|  |  | 68 | -12 | -16 | G_Temporal_Mid-191 |
| Deactivation | Cluster |  |  |  |  |
|  |  | -62 | -20 | -18 | G_Temporal_Mid-292 |
|  |  | -52 | -8 | 44 | S_Rolando-133 |
|  |  | -28 | -38 | -18 | G_ParaHippocampal-2136 |
|  |  | -24 | -32 | 66 | S_Rolando-436 |
|  |  | -52 | -8 | 44 | S_Rolando-133 |
|  |  | -28 | -38 | -18 | G_ParaHippocampal-2163 |
|  |  | -24 | -32 | 66 | S_Rolando-436 |

Activation and Deactivation during task conduction

Face Emotion


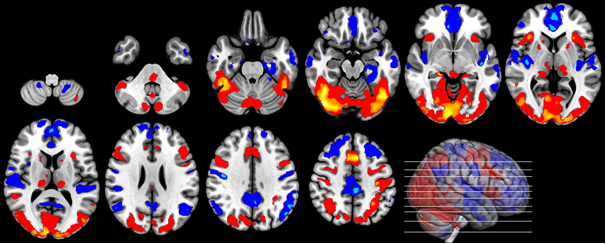


**Figure S1**. Activation and deactivation pattern of the Face Emotion task at basline.

Activation and Deactivation during task conduction

**Table S2**

Coordinates for Face Emotion task.

| Face Emotion |  | MNI coordinates | | | Anatomical brain region (MRIcroGL AICHA REF) |
| --- | --- | --- | --- | --- | --- |
| Activation | Peak |  |  |  |  |
|  |  | -48 | -38 | -26 | G_Temporal_Inf-397 |
|  |  | -48 | -54 | -22 | G_Fusiform-4170 |
|  |  | -44 | -84 | -14 | G_Occipital_Lat-363 |
|  |  | -44 | -82 | -2 | G_Occipital_Lat-565 |
|  |  | -42 | -68 | 4 | G_Occipital_Inf-273 |
|  |  | -40 | 16 | 22 | S_Inf_Frontal-217 |
|  |  | -32 | -68 | -56 | Cerebellum |
|  |  | -12 | -98 | 2 | G_Calcarine-3153 |
|  |  | -12 | -82 | -40 | Cerebellum |
|  |  | -6 | 16 | 48 | G_Supp_Motor_Area-1112 |
|  |  | 18 | -6 | -18 | G_ParaHippocampal-1162 |
|  |  | 34 | 8 | -28 | G_Temporal_Pole_Mid-2103 |
|  |  | 48 | -46 | -20 | G_Fusiform-4170 |
|  |  | 56 | -18 | -6 | S_Sup_Temporal-287 |
|  |  | 66 | -40 | 18 | S_Sup_Temporal-489 |
| Activation | Cluster |  |  |  |  |
|  |  | 42 | 2 | 48 | S_Precentral-430 |
| Deactivation | Peak |  |  |  |  |
|  |  | 10 | 46 | -6 | S_Anterior_Rostral-1108 |
|  |  | 20 | 26 | 56 | S_Sup_Frontal-48 |
|  |  | 22 | 38 | 38 | S_Sup_Frontal-37 |
|  |  | 38 | -14 | 12 | G_Insula-posterior-179 |
|  |  | 38 | 26 | 42 | G_Frontal_Mid-414 |
|  |  | 40 | -12 | 6 | G_Insula-posterior-179 |
|  |  | 42 | -14 | 4 | G_Temporal_Sup-283 |
|  |  | 44 | 54 | 0 | G_Frontal_Mid_Orb-221 |
|  |  | 48 | -14 | 14 | G_Insula-posterior-179 |
|  |  | 60 | -2 | 16 | S_Rolando-133 |
| Deactivation | Cluster |  |  |  |  |
|  |  | -38 | -16 | 36 | S_Rolando-133 |
|  |  | -2 | 4 | 44 | S_Cingulate-3117 |
|  |  | 2 | -38 | 50 | S_Cingulate-6120 |
|  |  | 14 | -70 | 26 | S_Parietooccipital-6148 |
|  |  | 24 | -40 | -20 | Cerebellum |
|  |  | 48 | -70 | 40 | G_Angular-253 |
|  |  | 68 | -20 | -14 | G_Temporal_Mid-292 |

Blinding, Tolerability, Acceptability

**Table S3**

Blinding efficacy.

|  | Participant’s Guess, n (%) | | |  |
| --- | --- | --- | --- | --- |
| Assignment | Active tDCS | Sham tDCS | DNK | Total |
| Experimental Group | 4 (18.2 %) | 3 (13.6 %) | 4 (18.2 %) | 11 |
| Control Group | 0 (0.0 %) | 4 (18.2 %) | 7 (31.8 %) | 11 |
| Total | 4 | 7 | 11 | 22 |

Note: DNK = do not know; James’ BI = 0.63 [95% CI: 0.44 - 0.82]. Bang’s BI = 0.09 [-0.38 - 0.56] in experimental group and 0.36 [0.08 − 0.65] in control group.

Blinding, Tolerability, Acceptability

**Table S4**

tDCS side effects and timing of side effects.

|  | **Group** | |  | | |
| --- | --- | --- | --- | --- | --- |
|  | Active (n=11) | Sham (n=11) | *LRT - X-squared* | *df* | *p* |
| Burning sensation | 5 | 5 |  |  |  |
| Pain over the stimulation sites | 2 | 2 |  |  |  |
| Heat | 6 | 3 | 0.7521 | 1 | 0.3858 |
| Itching over the stimulation sites | 8 | 2 | 4.5833 | 1 | 0.0323 |
| Headache | 0 | 0 |  |  |  |
| Other | 3 | 1 | 0.3056 | 1 | 0.5804 |
| Begin of stimulation | 16 | 14 | 3.4583 | 1 | 0.0629 |
| Middle of stimulation | 12 | 1 | 5.8935 | 1 | 0.0152 |
| End of stimulation | 6 | 2 | 0.1883 | 1 | 0.6643 |

*Note: LRT = likelihood ratio test, *p < .05*
